# Recombinant production, purification, crystallization, and structure analysis of human transforming growth factor β2 in a new conformation

**DOI:** 10.1038/s41598-019-44943-4

**Published:** 2019-06-17

**Authors:** Laura del Amo-Maestro, Laura Marino-Puertas, Theodoros Goulas, F. Xavier Gomis-Rüth

**Affiliations:** Proteolysis Lab; Structural Biology Unit; “María-de-Maeztu” Unit of Excellence, Molecular Biology Institute of Barcelona (CSIC); Barcelona Science Park, c/Baldiri Reixac, 15-21, 08028 Barcelona, Catalonia Spain

**Keywords:** Structural biology, Expression systems

## Abstract

Transforming growth factor β is a disulfide-linked dimeric cytokine that occurs in three highly related isoforms (TGFβ1–TGFβ3) engaged in signaling functions through binding of cognate TGFβ receptors. To regulate this pathway, the cytokines are biosynthesized as inactive pro-TGFβs with an N-terminal latency-associated protein preceding the mature moieties. Due to their pleiotropic implications in physiology and pathology, TGFβs are privileged objects of *in vitro* studies. However, such studies have long been limited by the lack of efficient human recombinant expression systems of native, glycosylated, and homogenous proteins. Here, we developed pro-TGFβ2 production systems based on human Expi293F cells, which yielded >2 mg of pure histidine- or Strep-tagged protein per liter of cell culture. We assayed this material biophysically and in crystallization assays and obtained a different crystal form of mature TGFβ2, which adopted a conformation deviating from previous structures, with a distinct dimeric conformation that would require significant rearrangement for binding of TGFβ receptors. This new conformation may be reversibly adopted by a certain fraction of the mature TGβ2 population and represent a hitherto undescribed additional level of activity regulation of the mature growth factor once the latency-associated protein has been separated.

## Introduction

Transforming growth factors β (TGFβs) are members of a superfamily of conserved cytokines, which also includes bone morphogenetic proteins, activins, and inhibins^1–3^. TGFβs were discovered in the early 1980s^4^, and three close orthologs are found in mammals (TGFβ1, TGFβ2, and TGFβ3^5^). They are ubiquitously expressed and secreted throughout the body^2,6^, where they are pleiotropically engaged in the physiology of nearly all tissues and cell types after activation and binding to TGFβ receptors of type I, II and III (TGFR-I, -II and -III)^3,7–10^. TGFβs mainly act through the SMAD pathway, which features a family of genes similar to *Caenorhabditis* “small worm phenotype” and *Drosophila* “mothers against decapentaplegic”. This pathway entails sequential binding of TGFR-II and TGFR-I by TGFβs to form a ternary complex that triggers phosphorylation of the latter by the former^11,12^. Thus, TGFβs regulate intracellular processes including transcription, translation, microRNA biogenesis, protein synthesis, and post-translational modifications^1,13^, which cascade into roles in growth, proliferation, differentiation, plasticity, migration, and death of epithelial and endothelial cells, as well as lymphocytes^14^. TGFβs are essential in biological events such as embryogenesis, wound healing, and immunity^2^, but also in pathologies such as Marfan syndrome, Parkinson’s disease, AIDS, organ fibrosis, autoimmune diseases, atherosclerosis and hypertension, asthma, diabetes, rheumatoid arthritis, encephalomyelitis, colitis, and most epithelial cancers, including prostate, breast, lung, colorectal, pancreatic, gastric, and skin cancers^2,13,15–20^.

Human TGFβs are produced as latent glycosylated precursors (pro-TGFβs), which consist of an ~250-residue N-terminal pro-domain dubbed latency-associated protein (LAP) and a C-terminal ~110-residue mature growth-factor moiety (GF). Two such precursors are joined through disulfides in the homodimeric small latent complex. These complexes are disulfide-linked to a third protein, one of three latent TGFβ binding proteins, in the large latent complexes^21–23^. After secretion, these complexes are targeted to fibrillin-rich microfibrils as inactive species that are covalently bound by tissue transglutaminase to the extracellular matrix for storage. Finally, the large latent complexes are transformed into biologically active GFs by thrombospondin 1, reactive oxygen species, integrins, and/or peptidases such as furin and other related pro-protein convertases. Proteolytic cleavage by these enzymes severs the linker between GF and LAP, but the two proteins remain noncovalently associated until physically separated for function^2,10,13,24,25^. In this way, most TGFβ in the body is latent and sequestered within the extracellular matrix, although it is also found on the surface of immune cells or in granules of platelets and mast cells^2^. Thus, localization and compartmentalization ensure tight spatial and temporal regulation of the GFs^22^.

Although TGFβ GFs are very similar (70–82% sequence identity in humans) and have partially overlapping functions, they also have distinct roles. This is reflected in their differential expression during embryogenesis and specific roles in renal fibrogenesis and regulation of airway inflammation and remodeling^26^. In particular, human TGFβ2 is biosynthesized as a latent 394-residue precursor (pro-TGFβ2) arranged as a glycosylated homodimer of 91 kDa containing three glycan chains attached to each LAP. Peptidolytic activation at bond R^302^-A^303^ (see UniProt [UP] entry P61812 for residue numbers of human TGFβ2 in superscripts^27,28^) produces the GF, which spans 112 residues and is arranged as a disulfide-linked 25-kDa homodimer noncovalently associated with the respective LAP moieties. Given the importance of this cytokine and its potential in therapeutic applications^29,30^, here we developed a new high-yield human expression system for tagged pro-TGFβ2. We report the X-ray crystal structure of its GF, which was obtained in a different crystallographic space group and deviates from current structures.

## Results and Discussion

### A new recombinant overexpression system for pro-TGFβ2

After its discovery, TGFβ2 GF was initially purified from human and porcine platelets^31,32^ and from bovine bone^33^. However, to obtain large amounts, recombinant expression systems are generally the option of choice as the resulting homogeneity and purity is greater than that of proteins purified from tissues or fluids. Mature human and mouse TGFβ2 GFs were obtained in high yields (~8–10 mg/liter of cell culture) from *Escherichia coli* systems^34^. However, these proteins lacked glycosylated LAP, which has (glycan-mediated) functions beyond latency maintenance^35–38^. In addition, these systems produced insoluble protein in inclusion bodies that had to be refolded^34,39^. From a functional perspective, mammalian expression systems are preferred for human cytokines as they provide native environments for folding and disulfide formation in the endoplasmic reticulum, glycosylation in the Golgi, and subsequent quality control in the endoplasmic reticulum. These factors ensure that only correctly folded proteins are secreted. However, the development of such systems has proven difficult for TGFβs^29,34,40,41^. Most initial trials—based on murine CHO and human HEK293 cells—outputted well below ~1 mg of pure protein per liter of cell culture, owing to low expression levels and multiple purification steps^42–45^. It was not until 2006 that Zou and Sun reported expression of pro-TGFβ2 from stable CHO-lec cell lines with a significantly higher yield^40^. However, this system was based on stable cell lines, which generally require long selection processes for establishment, are prone to contamination, require more time for preparation and for protein expression, and have little flexibility with respect to the constructs tested. In addition, the lack of equivalence of recombinant proteins produced in CHO and HEK cells owing to differences in the glycosylation patterns between hamsters and humans has been widely documented^46–48^.

**Figure 1:**
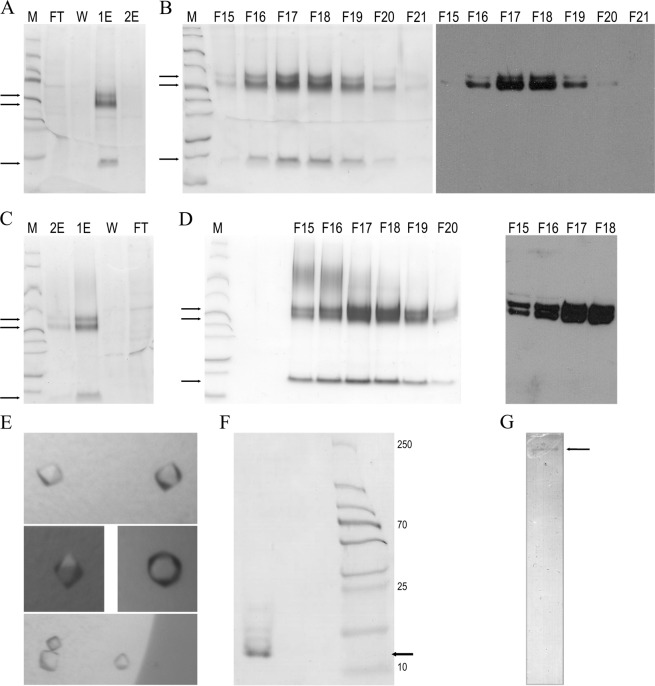
Production, purification and crystallization of human TGFβ2. (**A**) Reducing SDS-PAGE depicting N-terminally octahistidine-tagged pro-TGFβ2 after Ni-NTA affinity purification. M, molecular mass marker; FT, flow-through; W, wash step; 1E, first elution; and 2E, second elution. Black arrows pinpoint (*top to bottom*) intact pro-TGFβ2, LAP, and the GF in lane 1E. (**B**) Reducing SDS-PAGE of fractions (F15-F21) of the size-exclusion chromatography purification step (*left panel*) and Western-blot analysis of fractions F15-F21 employing an anti-histidine-tag antibody (*right panel*). (**C**,**D**), same as (**A**,**B**) for N-terminally Strep-tagged pro-TGFβ2. In (D), an anti-Strep-tag antibody was used. (**E**) Representative tetragonal crystals of mature TGFβ2 of ~20 microns maximal dimension. (**F**) Reducing SDS-PAGE of ~90 collected, carefully washed and dissolved diffraction-grade crystals revealing they contain mature TGFβ2 (black arrow). (**G**) Gelatin zymogram of pooled and purified crystallization drop supernatant showing a band pinpointed by an arrow corresponding to the mass of human α_2_-macroglobulin associated with gelatinolytic activity.The original gels used for panels A-D, F and G can be found in the SupplementaryInformation.

Here, we developed a new homologous production procedure for recombinant full-length human pro-TGFβ2 based on transient transfection of human Expi293F cells, a HEK293 cell-line variant that was adapted to high-density suspension growth and selected for high transfection efficiency and protein expression. Such transient expression systems are flexible, the constructs can be changed and retested fast, they have comparable yields to the stable systems, and they can be much more easily shared among scientists by just sending the plasmid^49^. We obtained ~2.7 and ~2.3 mg of N-terminally octahistidine-tagged and Strep-tagged forms, respectively, per liter of cell culture in only three days (Fig. 1A–D). The protein was in a disulfide-linked dimeric state, and a major fraction was cleaved at the inter-domain linker due to endogenous proteolytic processing, as previously reported for this and other TGFβs^34,45^. However, the LAP and the GF remained associated in size-exclusion chromatography and native polyacrylamide gel electrophoresis (PAGE). Our efforts to separate them by size-exclusion chromatography in the presence of high salt contents (1 M sodium chloride), chaotropic agents (1 M/4 M urea), detergents (0.05% sodium dodecylsulfate [SDS]), reducing agents (tris[2-carboxyethyl]phosphine or 1,4-dithiothreitol), and low-pH buffers (glycine pH 3.0) failed. We could not isolate them either by ionic exchange chromatography with 20 mM sodium acetate pH 4.0 as buffer and a sodium chloride gradient. This is consistent with previous reports on purified and recombinant pro-TGFβ1^45,50^ and with the very high affinity of GF and LAP for each other, with dissociation constant values in the low nanomolar range^51^. In contrast, other publications reported activation of TGFβs *in vitro* but only under harsh conditions including extreme pH values, high temperature, presence of peptidases and glucosidases, and presence of SDS and urea^34,40,52,53^.

### Separation and crystallization of mature TGFβ2

During studies of the interactions between induced human α_2_-macroglobulin (hα_2_M), a ~720-kDa homotetrameric pan-peptidase inhibitor purified from blood, and recombinant human pro-TGFβ2 (Marino-Puertas, del Amo-Maestro, Taulés, Gomis-Rüth & Goulas, manuscript in preparation), a mixture of both proteins was set up for crystallization. Diffraction-grade protein crystals appeared after five days with 20% isopropanol, 0.2 M calcium chloride, and 0.1 M sodium acetate pH 4.6 as reservoir solution (Fig. 1E). Reducing SDS-PAGE (Fig. 1F) and peptide mass fingerprinting of carefully washed and dissolved crystals indicated that the crystallized species was the GF. The crystals belonged to a hitherto undescribed, tightly-packed tetragonal space group, diffracted to 2.0 Å resolution, and contained half a GF disulfide-linked dimer per asymmetric unit. Diffraction data processing statistics are provided in Table 1. These crystals also appeared when pro-TGFβ2 alone was subjected to similar crystallization conditions. In this case, the crystals diffracted to lower resolution, i.e. hα_2_M apparently played a favorable role as an additive for crystallization. To pursue this further, we collected the supernatant from crystallization drops that had given rise to crystals, purified it by size-exclusion chromatography, and assayed a fraction that migrated according to the mass of hα_2_M by gelatin zymography. We detected gelatinolytic activity (Fig. 1G), which points to a contaminant present in the purified hα_2_M sample. Accordingly, we conclude that the low pH of the crystallization assay, together with the crystallization process, achieved the separation of the LAP and GF moieties that we could not obtain by chromatography (see above). This process was probably facilitated by a peptidolytic contaminant present in the purified hα_2_M sample, as peptidases trapped within the induced tetrameric hα_2_M cage are known to still possess activity^54–56^.

**Table 1:** Crystallographic data.

Dataset	Mature TGFβ2
**Data processing**	
Space group	P4_1_2_1_2
Cell constants (a and c, in Å)	55.57, 70.57
Wavelength (Å)	1.0332
No. of measurements/unique reflections	192,672/7,919
Resolution range (Å)	70.6–2.00 (2.12–2.00) ^a^
Completeness (%)	100.0 (99.9)
R_merge_	0.070 (2.495)
R_meas_/CC^1/2^	0.072 (2.546)/1.000 (0.870)
Average intensity	23.4 (1.7)
B-Factor (Wilson) (Å^2^)/Aver. multiplicity	56.6/24.3 (24.8)
**Structure refinement**	
Resolution range used for refinement (Å)	43.7–2.00
No. of reflections used (test set)	7,511 (407)
Crystallographic R_factor_ (free R_factor_)	0.217 (0.253)
No. of protein residues/atoms/solvent molecules	112/890/23
Correlation coefficient F_obs_-F_calc_ with all reflections/test set	0.943/0.938
***Rmsd*** **from target values**	
Bonds (Å)/angles (°)	0.010/1.18
Average B-factors (Å^2^) (all/protein)	66.2/66.4
**All-atom contacts and geometry analysis** ^**b**^	
**Residues**	
in favored regions/outliers/all residues	102 (93%)/0/110
outlying rotamers/bonds/angles/chirality/planarity	4/0/0/0/0
All-atom clashscore	1.7

^a^Data processing values in parenthesis are for the outermost resolution shell. ^b^According to the wwPDB X-ray Structure Validation Report.

### Structure of mature TGFβ2

The crystal structure was solved by maximum likelihood-scored molecular replacement, which gave a unique solution for one GF per asymmetric unit at final Eulerian angles and fractional cell coordinates (α, β, γ, x, y, and z) 348.4, 21.1, 119.9, 0.127, 0.696, and −0.899. The initial values for the rotation/translation function Z-scores were 6.0/14.0 and the final log-likelihood gain was 132. Taken together, these values indicated that P4_1_2_1_2 was the correct space group, and the final model after model rebuilding and refinement contained residues A^303^-S^414^ and 23 solvent molecules. Segments P^351^-W^354^ and N^371^-S^377^ were flexible but clearly resolved in the final Fourier map for their main chains. Table 1 provides refinement and model validation statistics.

**Figure 2:**
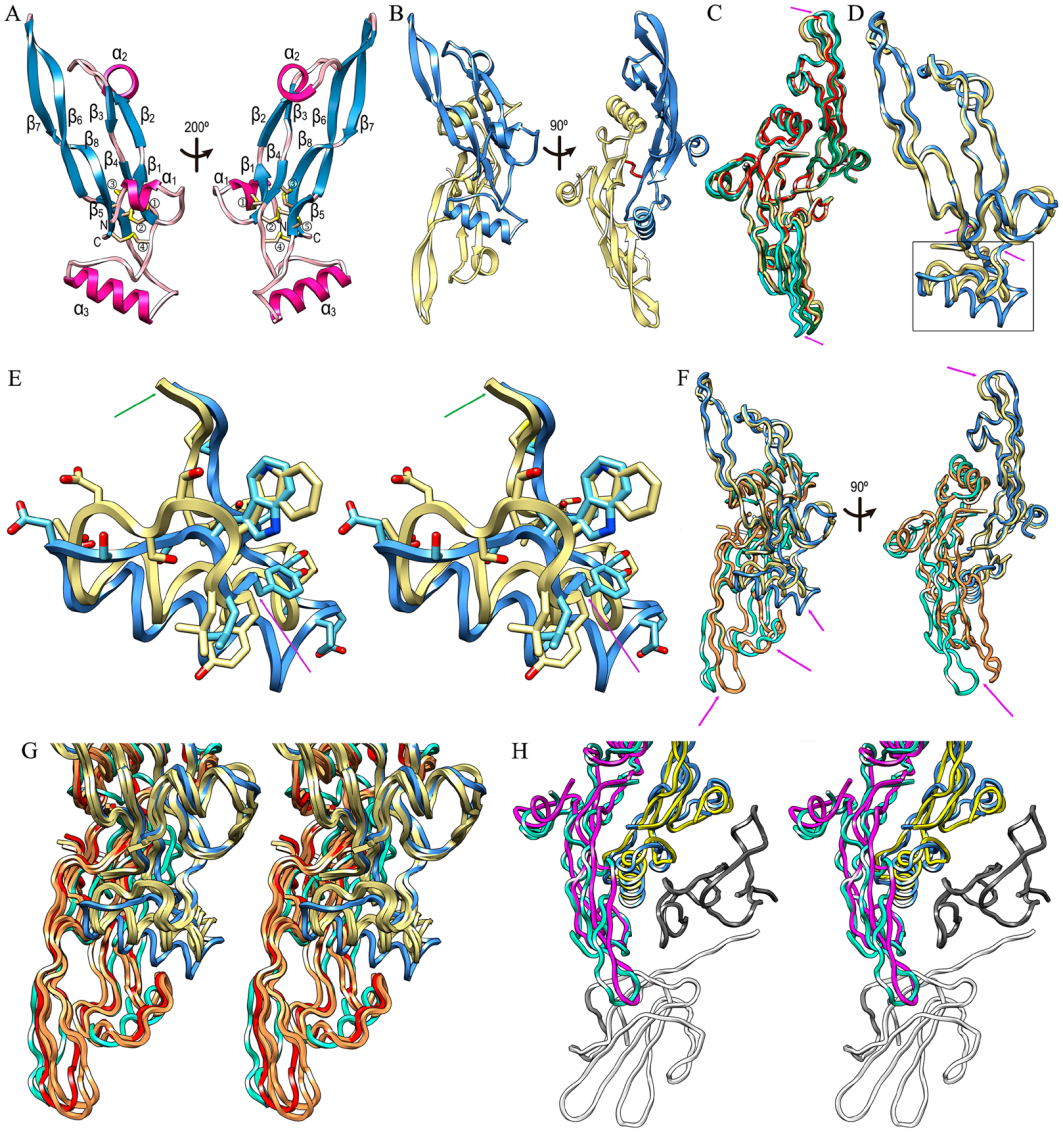
TGFβ2 in a new conformation. (**A**) Ribbon-type plot of human TGFβ2 (*left panel*) and after vertical rotation (*right panel*). The eight β-strands β1 (residues C^317^-R^320^), β2 (L^322^-D^325^), β3 (G^340^-N^342^), β4 (F^345^-A^347^), β5 (C^379^-S^382^), β6 (D^384^-I^394^), β7 (T^397^-S^404^), and β8 (M^406^-S^414^), as well as the three helices α1 (A^306^-F^310^), α2 (F^326^-L^330^), and α3 (Q^359^-I^370^), are labeled, as are the N- and the C-terminus. The four intramolecular disulfides are depicted for their side chains and labeled ① (C^309^-C^318^), ② (C^317^-C^380^), ③ (C^346^-C^411^) and ④ (C^350^-C^413^). The cysteine engaged in a symmetric intermolecular disulfide (C^379^) is further labeled as ⑤. (**B**) Human mature TGFβ2 dimer with one protomer in the orientation of A (*left panel*) in blue and the second in pale yellow (*left*), which is related to the former through a horizontal crystallographic twofold axis. An orthogonal view is provided in the *right panel*, the intermolecular disulfide is depicted as red sticks. (**C**) Superposition of the Cα-traces in ribbon presentation of the dimers of previously reported structures of mature TGFβ2 (PDB 2TGI, pale yellow; PDB 1TFG, red; PDB 4KXZ dimer AB, green; and PDB 4KXZ dimer DE, aquamarine) after optimal superposition of the respective top protomers. Magenta arrows pinpoint the only points of significant deviation, i.e. the tips of respective β-ribbons β6β7. The view is that of B (*right panel*). (**D**) Superposition of the protomers of PDB 2TGI in pale yellow onto the current structure (PDB 6I9J) in the view of A (*left panel*). The region of largest deviation (Y^352^-C^380^) is pinpointed by magenta arrows and framed. (**E**) Close-up in cross-eye stereo of the framed region of (**D**), with ribbon and carbons in pale yellow for PDB 2TGI and in blue/cyan for PDB 6I9J. Y^352^ and C^380^ are pinpointed by a magenta and a green arrow, respectively. (**F**) Superposition of the dimers of PDB 6I9J (top protomer in blue, bottom protomer in aquamarine) and PDB 2TGI (top protomer in pale yellow, bottom protomer in orange) in the views of (**B**). Owing to the different chain traces of segment Y^352^-C^380^ (top magenta arrow in the *left panel*, see also [D]), substantial variations are observed at distal regions of the bottom protomers. (**G**) Close-up in stereo of F (*left panel*) depicting PDB 6I9J (dimer in blue/aquamarine); PDB 2TGI and 4KXZ dimer AB, both in pale yellow/orange; and human TGFβ3 as found in its complex with the ectodomains of human TGFR-I and -II (PDB 2PJY; protomers in pale yellow and red). (**H**) Superposition in stereo of the dimers of PDB 6I9J (dimer in blue/aquamarine) and human TGFβ3 (PDB 2PJY dimer in yellow/magenta) in complex with the ectodomains of human TGFR-I (dark grey) and -II (white) in the orientation of B (*right panel*). TGFβ2 in PDB 6I9J must rearrange to bind the receptors as performed by TGFβ3.

Human TGFβ2 GF is an elongated α/β-fold molecule consisting of an N-terminal helix α1 disemboguing into a fourfold antiparallel sheet of simple up-and-down connectivity (Fig. 2A). Each strand is subdivided into two, β1 + β2, β3 + β4, β5 + β6, and β7 + β8, and the sheet is slightly curled backwards with respect to helix α1. The most exposed segment of the moiety is the tip of hairpin β6β7, and β-ribbon β2β3 is linked by a second α-helix (α2) as part of a loop, whose conformation is mediated by the *cis* conformation of residue P^338^. Finally, a long loop segment connects strands β4 and β5 and contains helix α3, whose axis is roughly perpendicular to the β-sheet. TGFβ2 is internally crosslinked by four disulfides forming a cysteine knot (Fig. 2A) and a further intermolecular disulfide by symmetric C^379^ residues links two crystallographic symmetry mates to yield the functional dimer. Here, helix α3 of one protomer nestles into the concave face of the β-sheet of the other protomer (Fig. 2B). The respective tips of hairpins β6β7, as well as β-ribbons β2β3 with connecting helices α2, are exposed for functional interactions.

### Comparison with previous TGFβ2 structures

The structure of isolated human TGFβ2 GF was solved in 1992 by two groups simultaneously. They obtained the same trigonal crystal form with half a disulfide-linked dimer per asymmetric unit (Protein Data Bank access codes [PDB] 1TFG^57^ and 2TGI^58^, see Table 2). In 2014, the complex of the GF with the Fab fragment of a neutralizing antibody was reported in an orthorhombic crystal form (PDB 4KXZ;^26^). These crystals contained two GF dimers per asymmetric unit, each bound to two Fab moieties. Three more structures of TGFβ2 GF were reported in 2017^59^ (Table 2). These corresponded to engineered monomeric forms from human (PDB 5TX4; in complex with TGFR-II ectodomain) and mouse (PDB 5TX2 and 5TX6) that lacked helix α3 and encompassed several point mutations.

**Table 2:** Crystal structures of TGFβ2.

PDB	Resolution (Å)	Residues ^a^	No. of residues	No. of copies in a.u.	State (Isolated/Complex)	Space group and cell constants (Å/°)	Organism	Reference	*rmsd* (Å) over residues^f^
1TFG	1.95	303–414	112	1	I	P3_2_21 a = b = 60.60, c = 75.20	Human	^57^	2.2/111
2TGI	1.80	303–414	112	1	I	P3_2_21 a = b = 60.60, c = 75.30	Human	^58^	2.3/111
4KXZ	2.83	303–414	112	4	C	P2_1_2_1_2 a = 131.20, b = 359.68 c = 64.63	Human	^26^	2.0/111
5TX2	1.82	303–414	93^c^	2	I	C2 a = 99.46, b = 33.36 c = 54.13, β = 109.6	Mouse	^59^	1.3/91
5TX4	1.88	303–414	92^d^	1	C	P2_1_2_1_2_1_ a = 39.02, b = 70.77 c = 77.17	Human	^59^	1.2/91
5TX6	2.75	303–414	93^c^	3	I	P3_1_21 a = b = 81.74, c = 80.93	Mouse	^59^	0.8/88
5TY4	2.90^b^	317–413	65^e^	1	C	P2_1_2_1_2_1_ a = 41.53, b = 71.33 c = 79.51	Human	^86^	0.8/65
6I9J	2.00	303–314	112	1	I	P4_1_2_1_2 a = b = 55.57, c = 70.57	Human	This work	—

^a^See UP entries P61812 and P27090 for human and mouse TGFβ2 sequences, respectively. ^b^Obtained by crystal electron diffraction. ^c^Mutant Δ354–373, K327R, R328K, L353R, A376K, C379S, L391V, Ι394V, Κ396R, Τ397Κ, and Ι400V. Contains an extra M at the N-terminus. ^d^Mutant Δ354–373, K327R, R328K, L353R, A376K, C379S, L391V, Ι394V, Κ396R, Τ397Κ, and Ι400V. ^e^Mutant Δ354–378, K327R, and R328K. ^f^Computed for the common Cα atoms of a protomer with the DALI program^84^ with respect to 6I9J.

Superposition of the GF structures of PDB 2TGI, 1TFG, and 4KXZ (Fig. 2C) revealed very similar conformations of bound and unbound protomers and dimers, despite distinct chemical and crystallographic environments, which can generally contribute to different conformations owing to crystallographic artifacts^60^. The *rmsd* values with respect to PDB 2TGI (considered hereafter the reference structure) upon superposition of one protomer were 0.26 Å (1TFG), 1.05 Å (4KXZ dimer AB), and 1.22 Å (4KXZ dimer DE) for 112, 111, and 111 common Cα atoms, respectively. Only minor variations occurred at the tips of hairpins β6β7 and at helix α2 (Fig. 2C). Interestingly, also the engineered monomeric variants kept the overall structure of the native TGFβ2 protomer, with the exception of a loop that replaced helix α3^59^. This close similarity was also found with the unbound human ortholog TGFβ3 (PDB 1TGJ^61^).

Inversely, the present GF structure (PDB 6I9J; hereafter the current structure) showed deviations from the reference structure, which are reflected by an *rmsd* of 1.90 Å for 107 common Cα atoms. The central β-sheets nicely coincide and only minor differences are found within the deviating regions of the above GF structures. However, a major rearrangement is found within segment Y^352^-C^380^, which encompasses helix α3 *plus* the flanking linkers to strands β4 and β5 (Fig. 2D,E). Owing to an outward movement of segment W^354^-D^357^, α3 is translated by maximally ~5.5 Å (at I^370^). This, in turn, causes downstream segment E^373^-C^379^ to be folded inward, with a maximal displacement of ~6.7 Å (at A^374^). This displacement cascades into a slight shift (~2 Å) of C^379^ and, thus, the intermolecular disulfide. These differences within protomers further result in variable arrangement of the dimers (Fig. 2F). The second protomer is rotated by ~10°, so that K^396^ at the tip of hairpin β6β7 is displaced by ~9 Å. In addition, the central β-sheets overlap but are laterally shifted with respect to each other.

**Figure 3:**
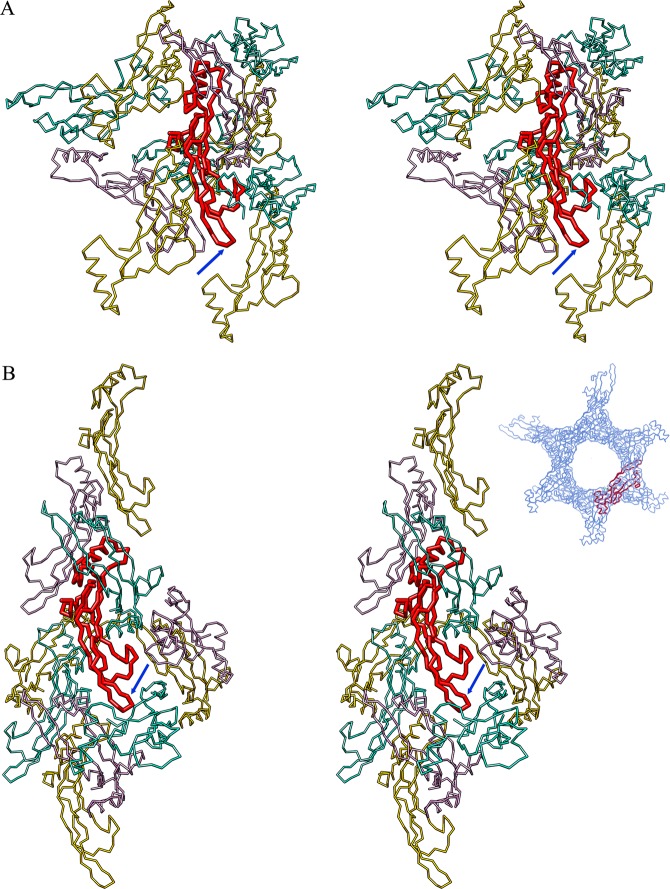
Crystal packing. (**A**) Cross-eye stereoplot showing the crystal packing of the current TGFβ2 structure (PDB 6I9J), with the protomer in the asymmetric unit in red and the surrounding symmetry mates in gold, plum and aquamarine. The tip of β-ribbon β6β7 is pinpointed by a blue arrow. (**B**) Same as (**A**) showing the crystal environment of the reference TGFβ2 structure (PDB 2TGI). The top right inset shows a view down the crystallographic threefold axis to illustrate the solvent channels. The protomer in the asymmetric unit is in red, the symmetry mates setting up the crystal lattice in blue.

Finally, inspection of the respective crystal packings of the current and reference structures (Fig. 3A,B) reveals that while the latter is loosely packed in a hexagonal honeycomb-like arrangement, with large void channels of > 40 Å diameter and high solvent content (61%), the former is tightly packed (solvent content of 43%). In particular, the tip of hairpin β6β7 is not engaged in significant crystal contacts in either crystal form, which supports that both conformations are authentic and do not result from crystallization artifacts.

### Potential implications of a new dimeric arrangement

Structural information on TGFR binding is available for human TGFβ1 and TGFβ3, whose wild-type forms were crystallized in complexes with the ectodomains of TGFR-I and -II (PDB 3KFD^62^; PDB 1KTZ^63^; and PDB 2PJY^12^). In the binary complex of TGFβ3 with TGFR-II (PDB 1KTZ), the cytokine was observed in a unique dimeric arrangement, termed “open”, in which the respective helices α3 were completely disordered. This led the second protomer to be rotated by ~180° and the tip of hairpin β6β7 to be displaced by ~36 Å when compared with the reference structure^63^. The functional significance of this structure, which differs from any other mature TGFβ dimer, is not clear.

By contrast, the other two complex structures, which contained both receptors, showed that the TGFβ1 and TGFβ3 isoforms dimerize very similarly to the unbound reference structure when bound to receptors (*rmsd* of 1.43 Å and 1.61 Å for 222 and 219 common Cα atoms for PDB 3KFD and PDB 2PJY, respectively). In contrast, they differed from the current structure (Fig. 2G), which shows a *rmsd* of 2.94 Å for 216 common Cα atoms when compared with the reference. In addition, both cytokine isoforms—and by similarity probably also TGFβ2—bound their receptors in an equivalent manner. TGFR-II was contacted by the tip of hairpin β6β7 and the linker between α2 and β3 of one protomer, and TGFR-I was liganded by the interface between protomers created by strands β5-β8 from one protomer and α1 *plus* the region around α3 of the other protomer. In addition, the TGFRs further contacted each other through the N-terminal extension of TGFR-II (Fig. 2H). Notably, the binding mode of TGFR-II was shared with the engineered monomeric variant of TGFβ2 (PDB 5TX4^59^). In contrast, superposition of the cytokine dimers of the current structure and the triple complexes evinced that among other structural elements hairpin β6β7 is displaced away from interacting segment S_52_-E_55_ of TGFR-II (see PDB 2PJY) by ~2.7 Å. This hairpin rearrangement also impairs interaction of the β5β8 ribbon with TGFR-I segment I_54_-F_60_. Thus, TGFRs could not bind the current structure in the same way they bind TGFβ1 and TGFβ3 without rearrangement.

### Conclusion

We developed a recombinant human overexpression system based on Expi293F cells grown in suspension, which produces high yields of well-folded human N-terminally histidine- or Strep-tagged pro-TGFβ2 with native post-translational modifications.

We further crystallized mature TGFβ2 in a different crystallographic space group, which deviates mainly in the region around helix α3 from current functional TGFβ1, TGFβ2 and TGFβ3 structures. Importantly, this region is the segment that shows the highest variability in sequence among TGFβs^26^. Although crystal packing artifacts cannot be completely ruled out, the fact that several different crystal forms of the three TGFβ GFs had produced highly similar structures to date suggests that the new conformation may be authentic and have functional implications.

The divergent conformation of the region around helix α3 led to differences in the dimer, which could not bind cognate TGFR-I and -II in the same way as TGFβ1 and TGFβ3 do. Moreover, as revealed by pro-TGFβ1 structures from pig (PDB 5VQF^64^) and human (PDB 5VQP^65^ and PDB 6GFF^66^), this region differed substantially between latent and mature moieties owing to the presence of the respective LAPs. Hence, the GF protomers associated differently within the respective dimers. Large differences were also found between the mature form and the pro-form of the more distantly related TGFβ family member activin A (PDB 2ARV^67^ and PDB 5HLY^68^). In contrast, bone morphogenetic factor 9 showed deviations in hairpin β6β7 but kept the dimer structure (PDB 5I05^69^ and PDB 4YCG^70^). Overall, we conclude that our mature structure may represent an inactive variant or be one of an ensemble of conformational states, which may still undergo an induced fit or selection fit mechanism to form a functional ternary ligand-receptor complex.

## Materials and Methods

### Protein production and purification

The coding sequence of human pro-TGFβ2 without its signal peptide, i.e. spanning the LAP (L^21^-R^302^) and the GF (A^303^-S^414^), was inserted in consecutive PCR steps into the Gateway pCMV-SPORT6 vector (ThermoFisher) in frame with the Kozak sequence. The signal peptide from the V-J2-C region of a mouse Ig κ-chain was used instead of the native leader sequence, as this was reported to be more efficient for expression^40^. This vector attached an N-terminal octahistidine-tag to the protein of interest. For the five PCR steps, oligonucleotides 5′-TCACCACCACCATCATCTCAGCCTGTCTACCTGCAGCA-3′; 5′-GGTTCCACTGGTGACCACCACCATCACCACCACCATC-3′; 5′-GGGTACTGCTGCTCTGGGTTCCAGGTTCCACTGGTGAC-3′; 5′-GACAGACACACTCCTGCTATGGGTACTGCTGCTC-3′; and 5′-CAATCCCGGGGCCACCATGGAGACAGACACACTCC-3′ were used as forward primers, respectively, and 5′-CAATCTCGAGCTAGCTGCATTTGCAAGACTTTAC-3′ was employed as the reverse primer. The intermediate PCR products were purified with the EZNA Cycle Pure Kit (Omega Bio-tek, USA) prior to the next step. The final PCR product was digested twice with *Sma*I and *Xho*I restriction enzymes (1 h at 37 °C and o/n at 37 °C), with an intercalated PCR product purification step. This product was ligated into pre-digested pCMV-SPORT6 by adding 2 μL of T4 DNA Ligase (ThermoFisher) per 20 μL of reaction (o/n at r.t.). The resulting plasmid (pS6-TGFB2-H8) was verified for its sequence (GATC Biotech) and transformed into competent *Escherichia coli* DH5α cells for vector storage and production.

Prior to transfection into mammalian cells, pS6-TGFB2-H8 was produced in *E*. *coli* DH5α, purified with the GeneJET Plasmid Maxiprep Kit (ThermoFisher) according to the manufacturer’s instructions, and stored in Milli-Q water at 1 mg/mL. Expi293F cells (ThermoFisher), which had been kept in suspension in FreeStyle F17 Expression Medium (Gibco) *plus* 0.2% Pluronic F-68 at 150 rpm in a humidified atmosphere with 8% CO_2_ in air at 37 °C, were transfected at a density of 1 × 10^6^ cells/mL with a mixture of 1 mg of purified pS6-TGFB2-H8 and 3 mg of linear 25-kDa polyethylenimine (Polysciences) in 20 mL of Opti-MEM Medium per liter. The mixture of the reagents was incubated for 15–20 min at room temperature and then added dropwise to the cells in 1 L-disposable Erlenmeyer flasks (Fisherbrand) after proper mixing. Cells were harvested for 72 h and then centrifuged at 2,800 × *g* in the JLA9.1000 rotor of an Avanti J-20XP centrifuge (Beckman Coulter) for 20 min, and the supernatant was subjected to single-step Ni-NTA affinity chromatography (washing buffer: 50 mM Tris·HCl pH 8.0, 250 mM sodium chloride, 20 mM imidazole; elution buffer: 50 mM Tris·HCl pH 8.0, 250 mM sodium chloride, 300 mM imidazole). Elutions were pooled, concentrated and subjected to size-exclusion chromatography in a Superdex 75 10/300 column (GE Healthcare) attached to an ÄKTA Purifier system (GE Healthcare) at r.t. with 20 mM Tris·HCl pH 8.0, 150 mM sodium chloride as buffer. Purified protein was routinely concentrated with Vivaspin centrifugal devices (Sartorius) with a 10-kDa cutoff, and concentrations were determined by A_280_ in a NanoDrop Microvolume spectrophotometer (ThermoFisher). Protein identity was confirmed by peptide mass fingerprinting and purity was assessed by 10% SDS-PAGE using Tris-Glycine buffer and Coomassie Brilliant Blue or silver staining, as well as by Western-blot analysis with a histidine antibody horse radish peroxidase conjugate (His-probe Antibody H-3 HRP, Santa Cruz Biotechnology) at 1:5,000 in PBS buffer further 0.1% in Tween 20.

To produce Strep-tagged pro-TGFβ2, plasmid pS6-TGFB2-H8 was modified by PCR to replace the histidine-tag with a twin Strep-tag with an intermediate G/S spacer (sequence WSHPQFEKGGGSGGGSGGSAWSHPQFEK) by using forward primer 5′-GGTGGAGGTTCTGGAGGTGGAAGTGGAGGTAGCGCATGGAGCCATCCACAATTCGAAAAGCTCAGCTGTCTACCTGC-3′ and reverse primer 5′-CTTTTCGAATTGTGGATGGCTCCAGTCACCAGTGGAACCTGGAACCCAGAGCAG-3′. The resulting PCR product was purified with the EZNA Cycle Pure Kit, phosphorylated with 1 μL of T4 polynucleotide kinase (ThermoFisher) per 20 μL of reaction (o/n at 37 °C), and ligated as above to generate plasmid pS6-TGFB2-STREP. This plasmid was further processed and transfected into Expi293F cells for protein production as above. Cells were harvested and centrifuged as aforementioned, and the supernatant was extensively dialyzed against 100 mM Tris·HCl pH 8.0, 150 mM sodium chloride and purified by single-step affinity chromatography using Strep-Tactin XT Superflow Suspension resin (iba) according to the manufacturer’s instructions (washing buffer: 100 mM Tris·HCl pH 8.0, 150 mM sodium chloride; elution buffer: 100 mM Tris·HCl pH 8.0, 150 mM sodium chloride, 50 mM biotin). Final polishing by size-exclusion chromatography, concentration, and purity assessment followed as above, except that Western-blot analysis was performed with a Streptavidin antibody horse radish peroxidase conjugate (Streptavidin-Peroxidase Antibody from *Streptomyces avidinii*; Sigma-Aldrich) at 1:1000 in PBS, supplemented with 0.1% Tween 20 and 1% BSA.

### Crystallization of mature TGFβ2 and diffraction data collection

Octahistidine-tagged pro-TGFβ2, mostly cleaved in the linker between LAP and GF, at ~5 mg/mL in 10 mM Tris·HCl pH 8.0, 150 sodium chloride was incubated o/n at 4 °C with human induced α_2_-macroglobulin (hα_2_M) at ~7 mg/mL in 10 mM Tris·HCl pH 8.0, 150 mM sodium chloride. The hα_2_M was previously purified from blood and reacted with methylamine as reported^71^. The hα_2_M/pro-TGFβ2 reaction mixture was subjected to crystallization assays by the sitting-drop vapor diffusion method at the Automated Crystallography Platform of Barcelona Science Park. Reservoir solutions were mixed by a Tecan robot and crystallization drops of 100 nL were dispensed by a Cartesian Microsys 4000 XL (Genomic Solutions) robot or a Phoenix nanodrop robot (Art Robbins) on 96 × 2-well MRC nanoplates (Innovadyne). Crystallization plates were stored at 20 °C or 4 °C in Bruker steady-temperature crystal farms, and successful conditions were scaled up to the microliter range in 24-well Cryschem crystallization dishes (Hampton Research).

Diffraction-grade crystals of ~20 microns maximal dimension were obtained at 20 °C with protein solution and 20% isopropanol, 0.2 M calcium chloride, 0.1 M sodium acetate pH 4.6 as reservoir solution from 1 or 2 μL: 1 μL drops. Carefully washed and pooled crystals revealed the presence of a species of ~12 kDa in SDS-PAGE, which was identified as TGFβ2 by peptide mass fingerprinting. Crystals were cryoprotected by immersion in reservoir solution *plus* 15% glycerol and flash cryocooled in liquid nitrogen. Diffraction data were collected at 100 K on a Pilatus 6 M pixel detector (Dectris) at beam line XALOC^72^ of the ALBA synchrotron in Cerdanyola (Catalonia, Spain). These data were processed with programs XDS^73^ and XSCALE^74^, and transformed with XDSCONV to MTZ format for the CCP4 suite of programs^75^. Crystals belonged to space group P4_1/3_2_1_2 based on the systematic extinctions, contained one mature TGFβ2 molecule per asymmetric unit (V_M_ = 2.14; 42.6% solvent contents according to^76^), and diffracted to 2.0 Å resolution.

### Proteolytic assay of crystallization-drop supernatant

Supernatant from crystallization drops that had produced crystals of mature TGFβ2, i.e. which contained methylamine-induced hα_2_M, was pooled and subjected to size-exclusion chromatography in a Superose6 10/300 column (GE Healthcare) at r.t. with 10 mM Tris·HCl pH 8.0, 150 mM sodium chloride as buffer. Fractions corresponding to hα_2_M (~720 kDa) were concentrated with Vivaspin centrifugal devices (Sartorius) with a 30-kDa cutoff and employed for zymography studies. To this aim, 10% SDS-PAGE gels were prepared containing 0.2% (w/v) gelatin. Protein samples were subjected to electrophoresis at 4 °C with equal volume of SDS-PAGE sample buffer without β-mercaptoethanol. Gels were washed twice in 20 mM Tris·HCl pH 7.4, 150 mM sodium chloride, 10 mM calcium chloride, 2.5% (v/v) Triton X-100 for 15 min and then incubated in the same buffer without detergent for 16 h at 37 °C under gentle shaking. Gels were stained with Coomassie Brilliant Blue.

### Structure solution and refinement

The structure of mature TGFβ2 was solved by likelihood-scoring molecular replacement with the PHASER^77^ program and the coordinates of a GF protomer crystallized in a different space group and unit cell (PDB 2TGI^58^). Subsequently, an automatic tracing step was performed with ARP/wARP^78^, which outputted a model that was completed through successive rounds of manual model building with the COOT program^79^ and crystallographic refinement with the PHENIX^80^ and BUSTER/TNT^81^ programs. The latter included TLS refinement.

### Miscellaneous

Structure figures were made with the CHIMERA program^82^. Structures were superposed with the SSM program^83^ within COOT. A search for structural similarity against the PDB was performed with DALI^84^. The final model of human TGFβ2 GF was validated with the wwPDB Validation Server (https://www.wwpdb.org/validation)^85^ and is available at the PDB at https://www.rcsb.org (access code 6I9J).

## Supplementary information

Supplementary information

## References

[1] Massague J (1990). The transforming growth factor-β family. Annu. Rev. Cell Biol..

[2] Jenkins G (2008). The role of proteases in transforming growth factor-β activation. Int. J. Biochem. Cell Biol..

[3] Derynck, R. & Budi, E. H. Specificity, versatility, and control of TGF-β family signaling. *Sci*. *Signal*. **12**, eaav5183 (2019). 10.1126/scisignal.aav5183 PMC6800142 30808818

[4] Frolik CA, Dart LL, Meyers CA, Smith DM, Sporn MB (1983). Purification and initial characterization of a type β transforming growth factor from human placenta. Proc. Natl. Acad. Sci. USA.

[5] Govinden R, Bhoola KD (2003). Genealogy, expression, and cellular function of transforming growth factor-β. Pharmacol. Ther..

[6] Travis MA, Sheppard D (2014). TGF-β activation and function in immunity. Annu. Rev. Immunol..

[7] Teicher BA (2001). Malignant cells, directors of the malignant process: role of transforming growth factor-β. Cancer Metastasis Rev..

[8] Akhurst RJ (2002). TGF-β antagonists: why suppress a tumor suppressor?. J. Clin. Invest..

[9] Hyytiainen M, Penttinen C, Keski-Oja J (2004). Latent TGF-β binding proteins: extracellular matrix association and roles in TGF-β activation. Crit. Rev. Clin. Lab. Sci..

[10] Principe DR (2014). TGF-β: duality of function between tumor prevention and carcinogenesis. J. Natl. Cancer Inst..

[11] Schmierer B, Hill CS (2007). TGFβ-SMAD signal transduction: molecular specificity and functional flexibility. Nat. Rev. Mol. Cell Biol..

[12] Groppe J (2008). Cooperative assembly of TGF-β superfamily signaling complexes is mediated by two disparate mechanisms and distinct modes of receptor binding. Mol. Cell.

[13] Li MO, Wan YY, Sanjabi S, Robertson AK, Flavell RA (2006). Transforming growth factor-β regulation of immune responses. Annu. Rev. Immunol..

[14] Ling TY, Huang YH, Lai MC, Huang SS, Huang JS (2003). Fatty acids modulate transforming growth factor-β activity and plasma clearance. FASEB J..

[15] Mishra L, Derynck R, Mishra B (2005). Transforming growth factor-β signaling in stem cells and cancer. Science.

[16] Massague J (2008). TGFβ in cancer. Cell.

[17] Padua D, Massague J (2009). Roles of TGFbeta in metastasis. Cell research.

[18] Theron AJ, Anderson R, Rossouw TM, Steel HC (2017). The role of Transforming Growth Factor β-1 in the progression of HIV/AIDS and development of non-AIDS-defining fibrotic disorders. Front. Immunol..

[19] Löffek S (2018). Transforming of the tumor microenvironment: implications for TGF-β inhibition in the context of immune-checkpoint therapy. J. Oncol..

[20] Ahmadi, A., Najafi, M., Farhood, B. & Mortezaee, K. Transforming growth factor-β signaling: tumorigenesis and targeting for cancer therapy. *J*. *Cell*. *Physiol*. **234**, 12173-12187 (2019). 10.1002/jcp.27955 PMC13484046 30537043

[21] Annes JP, Munger JS, Rifkin DB (2003). Making sense of latent TGFβ activation. J. Cell Sci..

[22] Rifkin DB (2005). Latent transforming growth factor-beta (TGF-β) binding proteins: orchestrators of TGF-β availability. J. Biol. Chem..

[23] Hinck, A. P., Mueller, T. D. & Springer, T. A. Structural biology and evolution of the TGF-β family. *Cold Spring Harb*. *Perspect*. *Biol*. **8**, a022103 (2016). 10.1101/cshperspect.a022103 PMC5131774 27638177

[24] Dubois CM, Laprise MH, Blanchette F, Gentry LE, Leduc R (1995). Processing of transforming growth factor β1 precursor by human furin convertase. J. Biol. Chem..

[25] Yang Z (2007). Absence of integrin-mediated TGFβ1 activation *in vivo* recapitulates the phenotype of TGFβ1-null mice. J. Cell Biol..

[26] Moulin A (2014). Structures of a pan-specific antagonist antibody complexed to different isoforms of TGFβ reveal structural plasticity of antibody-antigen interactions. Protein Sci..

[27] de Martin R (1987). Complementary DNA for human glioblastoma-derived T cell suppressor factor, a novel member of the transforming growth factor-β gene family. EMBO J..

[28] Marquardt H, Lioubin MN, Ikeda T (1987). Complete amino acid sequence of human transforming growth factor type β 2. J. Biol. Chem..

[29] Wilbers RH (2016). Co-expression of the protease furin in *Nicotiana benthamiana* leads to efficient processing of latent transforming growth factor-β1 into a biologically active protein. Plant Biotechnol. J..

[30] Soleimani Atena, Khazaei Majid, Ferns Gordon A, Ryzhikov Mikhail, Avan Amir, Hassanian Seyed Mahdi (2019). Role of TGF‐β signaling regulatory microRNAs in the pathogenesis of colorectal cancer. Journal of Cellular Physiology.

[31] Pircher R, Jullien P, Lawrence DA (1986). β-Transforming growth factor is stored in human blood platelets as a latent high molecular weight complex. Biochem. Biophys. Res. Commun..

[32] Cheifetz S (1987). The transforming growth factor-β system, a complex pattern of cross-reactive ligands and receptors. Cell.

[33] Ogawa Y, Seyedin SM (1991). Purification of transforming growth factors β1 and β2 from bovine bone and cell culture assays. Methods Enzymol..

[34] Huang T, Hinck AP (2016). Production, isolation, and structural analysis of ligands and receptors of the TGF-β superfamily. Methods Mol. Biol..

[35] Thomas GJ, Hart IR, Speight PM, Marshall JF (2002). Binding of TGF-β1 latency-associated peptide (LAP) to αvβ6 integrin modulates behaviour of squamous carcinoma cells. Br. J. Cancer.

[36] Nomura K, Tada H, Kuboki K, Inokuchi T (2002). Transforming growth factor-β-1 latency-associated peptide and soluble β-glycan prevent a glucose-induced increase in fibronectin production in cultured human mesangial cells. Nephron.

[37] Ali NA (2008). Latency associated peptide has *in vitro* and *in vivo* immune effects independent of TGF-β1. PloS ONE.

[38] Lee MJ (2013). Heparin inhibits activation of latent transforming growth factor-β1. Pharmacology.

[39] Schlunegger MP (1992). Crystallization and preliminary X-ray analysis of recombinant human transforming growth factor β2. FEBS Lett..

[40] Zou Z, Sun PD (2006). An improved recombinant mammalian cell expression system for human transforming growth factor-β2 and -β3 preparations. Prot. Expr. Purif..

[41] Bourdrel L (1993). Recombinant human transforming growth factor-β1: expression by Chinese hamster ovary cells, isolation, and characterization. Protein Expr. Purif..

[42] Madisen L (1989). Expression and characterization of recombinant TGF-β2 proteins produced in mammalian cells. DNA.

[43] Graycar JL (1989). Human transforming growth factor-β3: recombinant expression, purification, and biological activities in comparison with transforming growth factors-β1 and -β2. Mol. Endocrinol..

[44] Madisen L, Lioubin MN, Marquardt H, Purchio AF (1990). High-level expression of TGF-β2 and the TGF-β2(414) precursor in Chinese hamster ovary cells. Growth Factors.

[45] Gentry LE, Lioubin MN, Purchio AF, Marquardt H (1988). Molecular events in the processing of recombinant type 1 pre-pro-transforming growth factor β to the mature polypeptide. Mol. Cell Biol..

[46] Croset A (2012). Differences in the glycosylation of recombinant proteins expressed in HEK and CHO cells. J. Biotechnol..

[47] van den Nieuwenhof IM (2000). Recombinant glycodelin carrying the same type of glycan structures as contraceptive glycodelin-A can be produced in human kidney 293 cells but not in chinese hamster ovary cells. Eur. J. Biochem..

[48] Gaudry JP (2008). Purification of the extracellular domain of the membrane protein GlialCAM expressed in HEK and CHO cells and comparison of the glycosylation. Protein Expr. Purif..

[49] Geisse S, Voedisch B (2012). Transient expression technologies: past, present, and future. Methods Mol. Biol..

[50] Huber D, Fontana A, Bodmer S (1991). Activation of human platelet-derived latent transforming growth factor-β1 by human glioblastoma cells. Comparison with proteolytic and glycosidic enzymes. Biochem. J..

[51] Miller DM (1992). Characterization of the binding of transforming growth factor-β1, -β2, and -β3 to recombinant β1-latency-associated peptide. Mol. Endocrinol..

[52] Brown PD, Wakefield LM, Levinson AD, Sporn MB (1990). Physicochemical activation of recombinant latent transforming growth factor-beta’s 1, 2, and 3. Growth Factors.

[53] Munger JS (1997). Latent transforming growth factor-β: structural features and mechanisms of activation. Kidney Int..

[54] Barrett AJ, Starkey PM (1973). The interaction of α_2_-macroglobulin with proteinases. Characteristics and specificity of the reaction, and a hypothesis concerning its molecular mechanism. Biochem. J..

[55] Sottrup-Jensen L (1989). α-Macroglobulins: structure, shape, and mechanism of proteinase complex formation. J. Biol. Chem..

[56] Marrero A (2012). The crystal structure of human α_2_-macroglobulin reveals a unique molecular cage. Angew. Chem. Int. Ed..

[57] Schlunegger MP, Grütter MG (1992). An unusual feature revealed by the crystal structure at 2.2 Å resolution of human transforming growth factor-β2. Nature.

[58] Daopin S, Piez KA, Ogawa Y, Davies DR (1992). Crystal structure of transforming growth factor-β 2: an unusual fold for the superfamily. Science.

[59] Kim SK (2017). An engineered transforming growth factor beta (TGF-β) monomer that functions as a dominant negative to block TGF-β signaling. J. Biol. Chem..

[60] Janin J, Rodier F (1995). Protein-protein interaction at crystal contacts. Proteins.

[61] Mittl PR (1996). The crystal structure of TGF-β3 and comparison to TGF-β2: implications for receptor binding. Protein Sci..

[62] Radaev S (2010). Ternary complex of transforming growth factor-β1 reveals isoform-specific ligand recognition and receptor recruitment in the superfamily. J. Biol. Chem..

[63] Hart PJ (2002). Crystal structure of the human TβR2 ectodomain–TGF-β3 complex. Nat. Struct. Biol..

[64] Shi M (2011). Latent TGF-β structure and activation. Nature.

[65] Zhao B, Xu S, Dong X, Lu C, Springer TA (2018). Prodomain-growth factor swapping in the structure of pro-TGF-β1. J. Biol. Chem..

[66] Lienart S (2018). Structural basis of latent TGF-β1 presentation and activation by GARP on human regulatory T cells. Science.

[67] Harrington AE (2006). Structural basis for the inhibition of activin signalling by follistatin. EMBO J..

[68] Wang X, Fischer G, Hyvonen M (2016). Structure and activation of pro-activin A. Nat. Commun..

[69] Saito T (2017). Structural basis of the human endoglin-BMP9 interaction: insights into BMP signaling and HHT1. Cell Rep..

[70] Mi L-Z (2015). Structure of bone morphogenetic protein 9 procomplex. Proc. Natl. Acad. Sci. USA.

[71] Goulas, T., Garcia-Ferrer, I., García-Piqué, S., Sottrup-Jensen, L. & Gomis-Rüth, F. X. Crystallization and preliminary X-ray diffraction analysis of eukaryotic α_2_ -macroglobulin family members modified by methylamine, proteases and glycosidases. *Mol. Oral Microbiol.* **29**, 354–364 (2014). 10.1111/omi.12069 25052482

[72] Juanhuix J (2014). Developments in optics and performance at BL13-XALOC, the macromolecular crystallography beamline at the ALBA synchrotron. J. Synchrotron Radiat..

[73] Kabsch W (2010). XDS. Acta Crystallogr. sect. D.

[74] Kabsch W (2010). Integration, scaling, space-group assignment and post-refinement. *Acta Crystallogr*. *sect*. D.

[75] Winn MD (2011). Overview of the CCP4 suite and current developments. Acta Crystallogr. sect. D.

[76] Matthews BW (1968). Solvent content of protein crystals. J. Mol. Biol..

[77] McCoy AJ (2007). Phaser crystallographic software. J. Appl. Crystallogr..

[78] Langer G, Cohen SX, Lamzin VS, Perrakis A (2008). Automated macromolecular model building for X-ray crystallography using ARP/wARP version 7. Nat. Protoc..

[79] Emsley P, Lohkamp B, Scott WG, Cowtan K (2010). Features and development of Coot. Acta Crystallogr. sect. D.

[80] Afonine PV (2012). Towards automated crystallographic structure refinement with phenix.refine. Acta Crystallogr. sect. D.

[81] Smart OS (2012). Exploiting structure similarity in refinement: automated NCS and target-structure restraints in BUSTER. Acta Crystallogr. sect. D.

[82] Pettersen, E. F. *et al*. UCSF Chimera - A visualization system for exploratory research and analysis. *J*. *Comput*. *Chem*. **25**, 1605–1612 (2004). 10.1002/jcc.20084 15264254

[83] Krissinel E, Henrick K (2004). Secondary-structure matching (SSM), a new tool for fast protein structure alignment in three dimensions. Acta Crystallogr. sect. D.

[84] Holm L, Laakso LM (2016). Dali server update. Nucleic Acids Res..

[85] Berman H, Henrick K, Nakamura H (2003). Announcing the worldwide Protein Data Bank. Nat. Struct. Biol..

[86] de la Cruz MJ (2017). Atomic-resolution structures from fragmented protein crystals with the cryoEM method MicroED. Nat. Methods.

