# Supplementary material for: Recombinant production, purification, crystallization, and structure analysis of human transforming growth factor β2 in a new conformation
Source: Sci Rep. 2019 Jun 17;9:8660. doi: 10.1038/s41598-019-44943-4 (PMC6572864; doi:10.1038/s41598-019-44943-4)
Supplement: Supplementary file 1 — Supplementary information [file 41598_2019_44943_MOESM1_ESM.pdf]

Recombinant production, purification, crystallization, and structure analysis of human transforming growth factor β2 in a new conformation

Laura del Amo-Maestro [#], Laura Marino-Puertas [#], Theodoros Goulas [*] & F. Xavier Gomis-Rüth [*]

Proteolysis Lab; Structural Biology Unit; "María-de-Maeztu" Unit of Excellence; Molecular Biology Institute of Barcelona (CSIC); Barcelona Science Park; c/Baldiri Reixac, 15-21; 08028 Barcelona (Catalonia, Spain).

SUPPLEMENTAL FIGURE FOR REVIEW ONLY

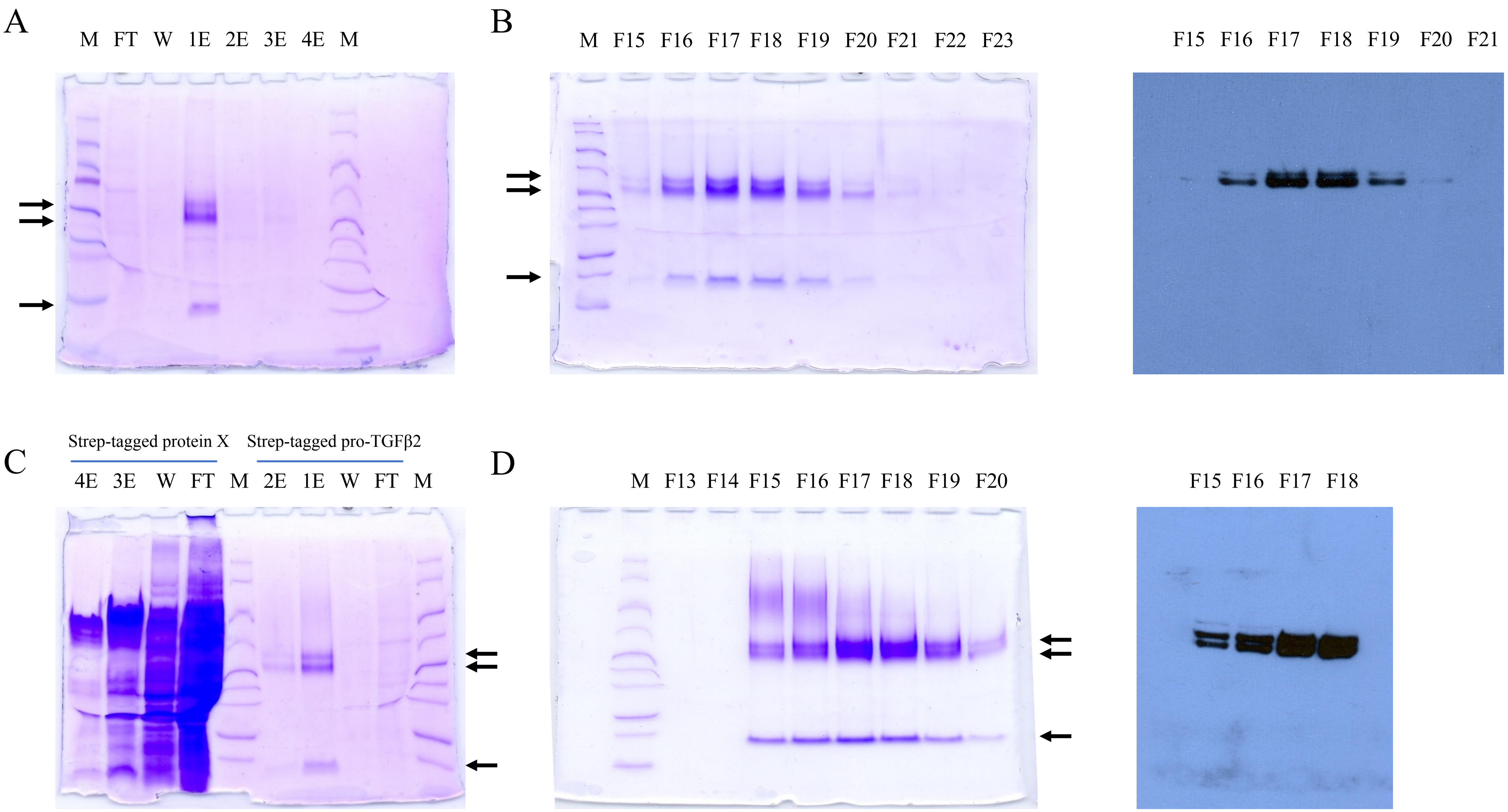
A
M FT W 1E 2E 3E 4E M
B
M F15 F16 F17 F18 F19 F20 F21 F22 F23
F15 F16 F17 F18 F19 F20 F21
C
Strep-tagged protein X
Strep-tagged pro-TGFβ2
4E 3E W FT M 2E 1E W FT M
D
M F13 F14 F15 F16 F17 F18 F19 F20
F15 F16 F17 F18

F

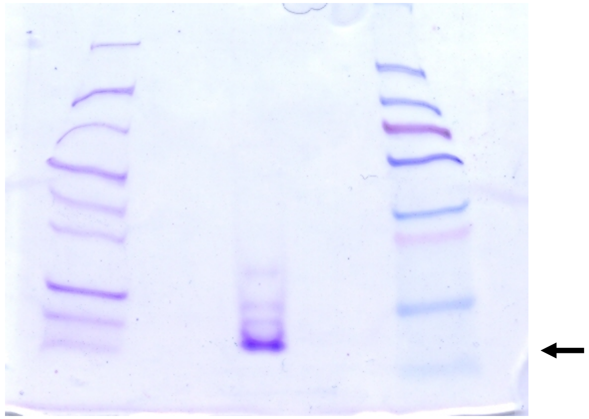

G

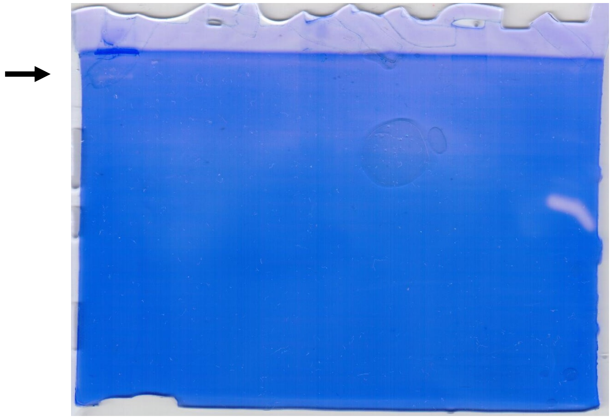

Supplement for Figure 1 showing the entire gels of the panels depicted — Production, purification and crystallization of human TGFβ2. **(A)** Reducing SDS-PAGE depicting N-terminally octahistidine-tagged pro-TGFβ2 after Ni-NTA affinity purification. M, molecular mass marker; FT, flow-through; W, wash step; 1E, first elution; 2E, second elution; 3E, third elution; and 4E, forth elution. Black arrows pinpoint (top to bottom) intact pro-TGFβ2, LAP, and the GF in lane 1E. **(B)** Reducing SDS-PAGE of fractions (F15-F23) of the size-exclusion chromatography purification step (left panel) and Western-blot analysis of fractions F15-F21 employing an anti-histidine-tag antibody (right panel). **(C)** and **(D)**, same as (A) and (B) for N-terminally Strep-tagged pro-TGFβ2. In (C) SDS-PAGE is shared with other Strep-tagged protein. In (D), an anti-Strep-tag antibody was used. **(E)** Representative tetragonal crystals of mature TGFβ2 of ~20 microns maximal dimension. **(F)** Reducing SDS-PAGE of ~90 collected, carefully washed and dissolved diffraction-grade crystals revealing they contain mature TGFβ2 (black arrow). **(G)** Gelatin zymogram of pooled and purified crystallization drop supernatant (*leftmost lane*) showing a band pinpointed by an arrow corresponding to the mass of human α2-macroglobulin associated with gelatinolytic activity. The white band on the right corresponds to the control (trypsin).
